# Opto-intelligence spectrometer using diffractive neural networks

**DOI:** 10.1515/nanoph-2024-0233

**Published:** 2024-07-02

**Authors:** Ze Wang, Hang Chen, Jianan Li, Tingfa Xu, Zejia Zhao, Zhengyang Duan, Sheng Gao, Xing Lin

**Affiliations:** School of Optics and Photonics, Beijing Institute of Technology, Beijing 100081, China; Department of Electronic Engineering, 12442 Tsinghua University, Beijing 100084, China; Key Laboratory of Photoelectronic Imaging Technology and System, Ministry of Education of China, Beijing 100081, China; Beijing Institute of Technology Chongqing Innovation Center, Chongqing 401135, China

**Keywords:** opto-intelligence spectrometer, photonic neural networks, spectral reconstruction

## Abstract

Spectral reconstruction, critical for understanding sample composition, is extensively applied in fields like remote sensing, geology, and medical imaging. However, existing spectral reconstruction methods require bulky equipment or complex electronic reconstruction algorithms, which limit the system’s performance and applications. This paper presents a novel flexible all-optical opto-intelligence spectrometer, termed OIS, using a diffractive neural network for high-precision spectral reconstruction, featuring low energy consumption and light-speed processing. Simulation experiments indicate that the OIS is able to achieve high-precision spectral reconstruction under spatially coherent and incoherent light sources without relying on any complex electronic algorithms, and integration with a simplified electrical calibration module can further improve the performance of OIS. To demonstrate the robustness of OIS, spectral reconstruction was also successfully conducted on real-world datasets. Our work provides a valuable reference for using diffractive neural networks in spectral interaction and perception, contributing to ongoing developments in photonic computing and machine learning.

## 1 Introduction

Photonic neural networks (PNNs) [1], [2], [3] utilize light’s principles for computations, achieving speed and efficiency far beyond traditional electronic neural networks. These networks exploit optical systems’ parallel processing and high bandwidth, significantly reducing energy consumption and latency for data processing, machine learning, and real-time analytics tasks. Within this innovative domain, diffractive neural networks (DNNs) have received extensive and continuous attention due to their simplicity, efficiency, strong generalization ability, and scalability [4], [5], [6], [7], [8], [9], [10], [11], [12], [13], [14]. Comprising several modulation layers that adjust the light field’s amplitude or phase, DNNs refine modulation elements through deep learning, facilitating arbitrary input-to-output mapping functions. This capability addresses various AI challenges at the speed of light, presenting a novel computational device solution for the post-Moore’s Law era.

Spectral reconstruction involves the utilization of instruments such as spectrometers to reconstruct the spectral information of substances from acquired data, offering profound insights into the composition, structure, and dynamics of samples [15]. This technology finds widespread application in various domains, including remote sensing [16], [17], geology [18], medical imaging [19], [20], and agriculture [21]. However, architectures based on existing design approaches often exhibit limited flexibility, particularly when tasked with considering on-demand, non-conventional responses [14]. Moreover, these systems are encumbered by cumbersome and complex structures, and some additionally require complex electronic reconstruction algorithms.

Here, we introduce a novel all-optical opto-intelligence spectrometer using diffractive neural networks, named OIS, for the spectral reconstruction of spatially coherent or incoherent multispectral input light sources. The OIS establishes a mapping relationship between input and output, transforming the spectral amplitude of the input light source into the detected intensity at the output plane, with each detector corresponding to a specific spectral band. Moreover, we optimize the phase of the modulation layers using two types of mean squared error (MSE) loss functions to not only reconstruct the input spectrum with high quality but also ensure a reasonable and high-contrast intensity distribution at the output plane. Initially, we validate the OIS’s capability for the spectral reconstruction of spatially coherent input samples through simulation experiments, achieving high-quality spectral reconstruction regardless of aperture size variations. Then, following the reference method [8], we introduce random phases at the input plane and simulate spatially incoherent inputs through multiple forward propagations and averaging at the output plane. The OIS still reconstructs spectra effectively, although the reconstruction quality gradually decreases as the aperture size increases. To address this problem, a simple yet effective electrical calibration module is designed at the backend of the optical reconstruction module to further enhance the quality of spectral reconstruction. After correction by the electrical calibration module, the reconstruction results closely match the inputs, with outcomes even surpassing those of spatially coherent spectral reconstruction tasks. To further validate the model’s generalizability, we successfully perform spectral reconstruction on real-world datasets, demonstrating the proposed architecture’s applicability in spectral reconstruction tasks and offering a novel approach for future spectral reconstruction endeavors.

## 2 Methods

### 2.1 Optical reconstruction

**Figure 1:**
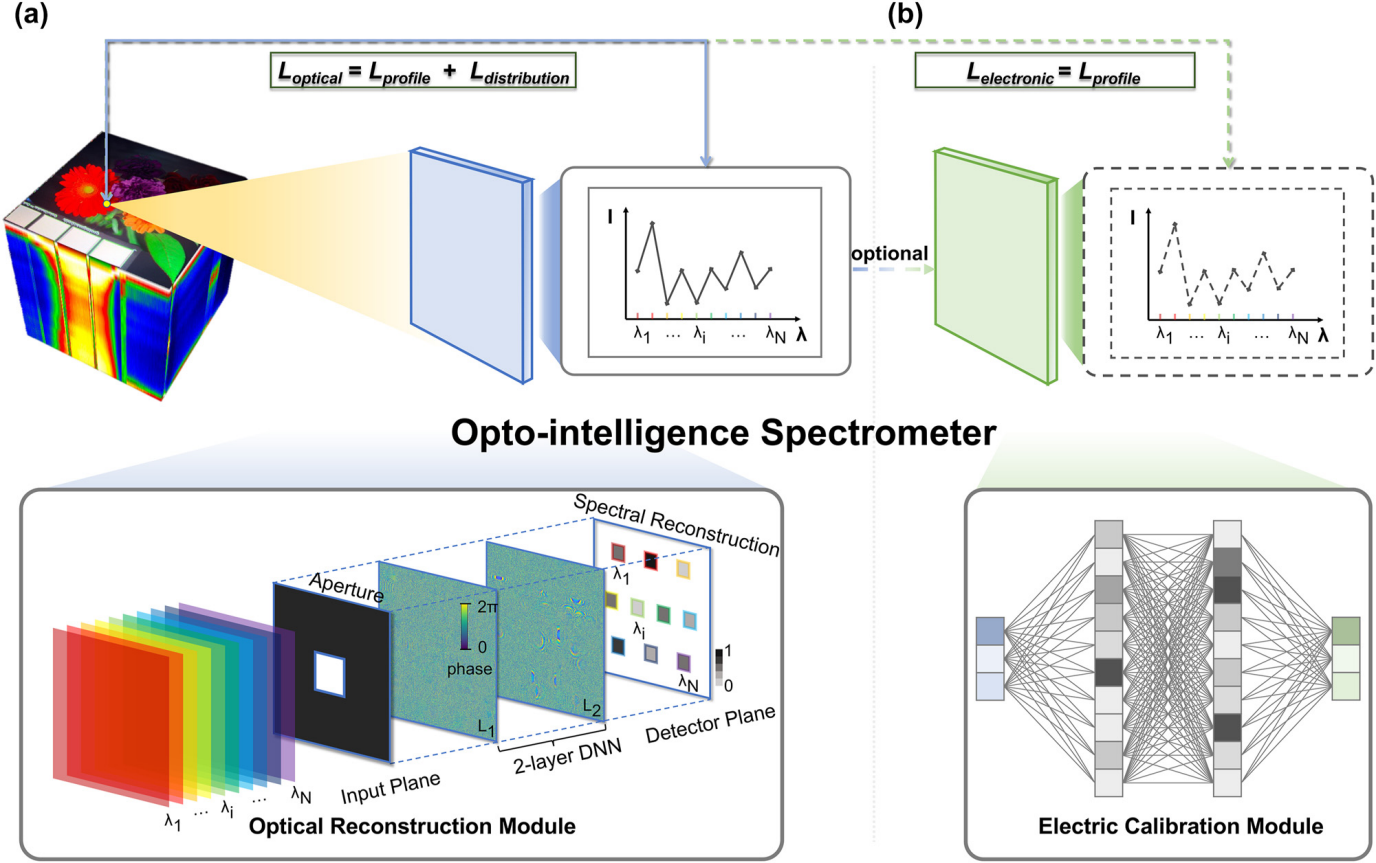
Spectral reconstruction architecture. (a) Optical reconstruction module: the architecture of opto-intelligence spectrometer using diffractive neural networks (OIS). The input plane light source comprises multiple spectral bands that are spatially coherent or incoherent. The spectral amplitude of each band is uniformly distributed across the plane and corresponds to a detector at the output plane. (b) Electrical calibration module: the architecture of electric neural network (ENN) for spectrum calibration. The module utilizes a symmetric fully-connected neural network with identical input and output dimensions. The input consists of the results from the optical reconstruction module, while the output delivers higher-quality spectral reconstruction results, refined through electrical calibration.

As demonstrated in Figure 1(a), the optical reconstruction module designed in this work leverages the OIS for the precise reconstruction of spectra. The input to the system is a surface light source, characterized by spatial coherence or spatial incoherence and encompassing multiple spectral bands (*λ*_*i*_, *i* = 1, …, *N*). Within the spatial domain, each channel of the light source exhibits uniform amplitude, with a fixed, uniform phase for spatial coherence and a random, undetermined phase for spatial incoherence. During the *k*th propagation, the input light field, upon traversing a rectangular aperture, is represented as follows:

(1) $$U_{\lambda_{i}}^{k}=rect\left(A_{\lambda_{i}}e^{j\cdot \varphi_{k}\left(x,y\right)}\right),$$

where $j=\sqrt{-1}$ . For the wavelength channel *λ*_*i*_, the amplitude $A_{\lambda_{i}}$ is uniformly distributed across the field and remains constant over time, corresponding to the spectral amplitude of that band. For the spatially coherent inputs, the phase $\varphi_{k}\left(x,y\right)$ is a real constant. For the spatially incoherent inputs, the phase $\varphi_{k}\left(x,y\right)$ at different spatial positions (*x*, *y*) undergoes random variations. The input light field is transformed by the OIS, resulting in an output light field distribution at the output plane, as illustrated by the following equation:

(2) $$O_{\lambda_{i}}^{k}={\left|M_{\lambda_{i}}U_{\lambda_{i}}^{k}\right|}^{2},$$

where $O_{\lambda_{i}}^{k}$ denotes the intensity distribution of the output light field for the *i*th channel during the *k*th propagation, and $M_{\lambda_{i}}$ represents the modulation effect of the OIS, employing phase-only modulation. For spatially coherent samples, the output remains constant over time. However, for spatially incoherent samples, the phase varies with time, leading to a corresponding variation in the output over time. During the training process, to simulate the effect of spatial incoherence, the average of multiple output light intensity distributions over several repeated forward propagation processes is calculated according to the principle of intensity superposition [8]. Since the modulation layers do not change over time, inputs at these forward propagation processes pass through the same modulation function. Let *N*_*tr*_ represent the number of repetitions for generating random phases during the training process. Then, the output intensity for the *λ*_*i*_ spectral band can be expressed as:

(3) $$\begin{array}{cc} O_{\lambda_{i}} & =\frac{1}{N_{tr}}\underset{k}{\sum }O_{\lambda_{i}}^{k}\\ & =\frac{1}{N_{tr}}\underset{k}{\sum }{\left|M_{\lambda_{i}}rect\left(A_{\lambda_{i}}e^{j\cdot \varphi_{k}\left(x,y\right)}\right)\right|}^{2}. \end{array}$$

In the context of a multi-wavelength DNN, the aggregate intensity distribution across various wavelengths can be mathematically described as the cumulative superposition of the detected intensity distributions corresponding to each individual wavelength [6]:

(4) $$\begin{array}{cc} O & =\underset{\lambda_{i}}{\sum }O_{\lambda_{i}}\\ & =\frac{1}{N_{tr}}\underset{\lambda_{i}}{\sum }\underset{k}{\sum }{\left|M_{\lambda_{i}}rect\left(A_{\lambda_{i}}e^{j\cdot \varphi_{k}\left(x,y\right)}\right)\right|}^{2}. \end{array}$$

Subsequently, *N* detectors are utilized to record the intensity information at specific positions. Following normalization, the results of spectral reconstruction are obtained as follows:

(5) $$\begin{array}{cc} A^{\prime } & =\left(A_{\lambda_{1}}^{\prime },A_{\lambda_{2}}^{\prime },\ldots ,A_{\lambda_{N}}^{\prime }\right)\\ & =Norm\left(I_{1},I_{2},\ldots ,I_{N}\right), \end{array}$$

where *A*′ represents the normalized vector of spectral intensities, while *I*_*i*_ denotes the intensity detected by the *i*th detector. To achieve optimal reconstruction results while also considering the distribution of light field energy, we employ two types of MSE loss for cooperative training:

(6) $$\begin{array}{cc} L_{\text{optical}} & =L_{\text{profile}}+L_{\text{distribution}}\\ & ={\left\|A^{\prime }-A\right\|}_{2}+{\left\|I-I_{gt}\right\|}_{2}, \end{array}$$

where *A* signifies the spectral amplitude labels corresponding to the input, whereas *I* represents the output light field distribution, with *I*_*gt*_ denoting the ground truth associated with this distribution.

### 2.2 Electric calibration

After the process of optical reconstruction, we have the foundational capability to reconstruct the spectral information of the input light source accurately. In pursuit of elevating the fidelity of these reconstructions, we integrate traditional electronic neural networks for electrical calibration at the posterior segment of the optical output pathway. It is noteworthy that this strategic incorporation is predicated upon the achievement of the pre-existing high caliber of optical reconstruction. Consequently, the deployment of a straightforward, fully connected neural network at this juncture is deemed sufficient to facilitate notable enhancements in the overall quality of the spectral reconstruction.

As illustrated in Figure 1(b), a symmetric architecture is adopted, with the dimensions of each layer (input dimension, output dimension) being (10, 64), (64, 64), and (64, 10), respectively. The spectral vector *A*′ resulting from optical reconstruction is corrected through the electronic neural network, yielding a new spectral intensity vector *A*′′. During the weight optimization process of the electronic neural network, the parameters of the optical modulation layers remain fixed. The update of the weights is guided by the following loss function:

(7) $$L_{\text{electronic}}={\left\|A^{\prime \prime }-A\right\|}_{2}.$$

## 3 Results

### 3.1 Spatially coherent optical reconstruction

Initially, we validate an OIS with two diffractive modulation layers through simulation experiments on the task of spatially coherent spectral reconstruction of ten spectral channels. The OIS was implemented using Python version 3.8.13 and PyTorch framework version 1.11.0. using a server (GeForce RTX 3090 Graphical Processing Unit, GPU, and Intel(R) Xeon(R) Gold 5218R CPU @2.10 GHz and 128 GB of RAM, running an Ubuntu 18.04 operating system). The custom spectral dataset spans a wavelength range of 400–500 nm, with ten bands selected at 10 nm intervals, namely 400 nm, 410 nm, 420 nm, 430 nm, 440 nm, 450 nm, 460 nm, 470 nm, 480 nm, and 490 nm. The spectral amplitudes for these ten bands are randomly generated within the range of 0–1. A total of 60,000 spectral sets are generated for the training dataset, and an additional 10,000 spectral sets for the testing dataset, with both being randomly shuffled. Accordingly, each of the ten detectors at the output plane is dedicated to capturing spectral information for a distinct wavelength. For network training, the Adam optimizer was employed to fine-tune the phase modulation coefficients of the optical diffractive elements, each sized at 4 μm × 4 μm. The phase modulation range of diffractive element is constrained between 0 and 2π, which we found that 4-bit quantization only slightly reduces the reconstruction performance.

We initiated our evaluation of the spectrum reconstruction capabilities of the OIS by setting the number of modulation elements per network layer to 800 × 800, which equates to a network layer size of 3.2 mm × 3.2 mm. Subsequent assessments compared network performance across various aperture sizes, referred to a rectangular aperture in the input plane shown in Figure 1(a) and the rectangular aperture sizes are denoted as *K* × *K*, where *K* = 160 μm, 320 μm, 400 μm, and 800 μm. The OIS was configured with two layers, and the inter-layer distance was optimized by leveraging the maximum half-cone diffraction angle theory [4], [5]. The training process was undertaken with a batch size of 8, starting with an initial learning rate of 0.01, which was halved after each epoch to facilitate convergence. The network training concluded after 10 epochs, successfully achieving the desired mapping function between inputs and outputs.

**Figure 2:**
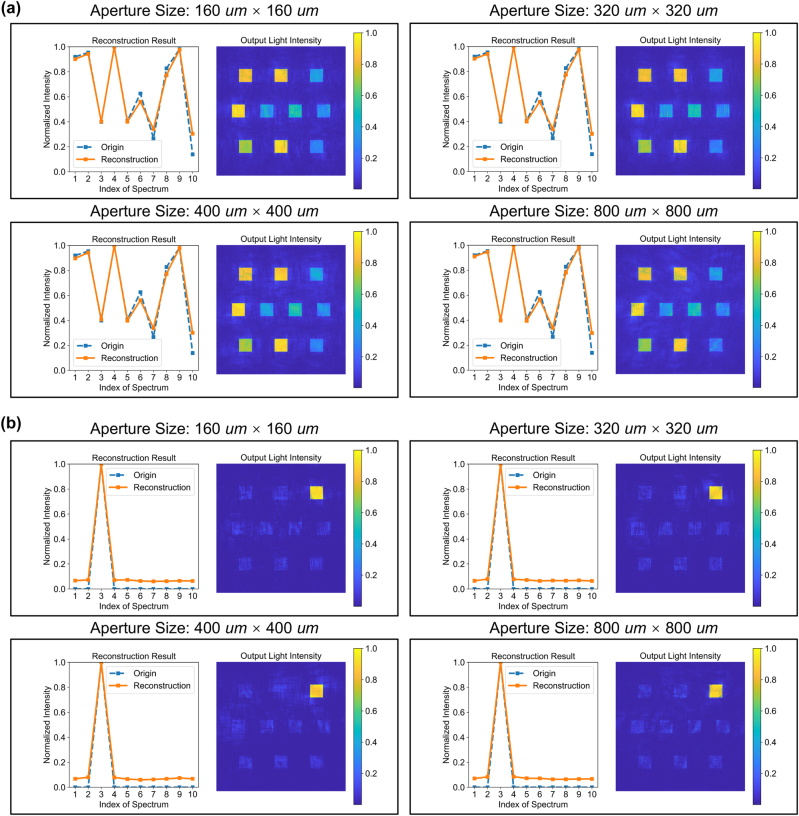
OIS for spatially coherent spectral reconstruction without electric calibration. Spectral resolution, 10 nm. (a) Left: The spectral amplitude distribution of ten spectral bands randomly generated along with the results of spectral reconstruction. Right: The intensity distribution at the output plane. (b) Left: The spectral amplitude distribution of the input containing only the 420 nm wavelength band, along with the results of spectral reconstruction. Right: The intensity distribution at the output plane.

The quantitative evaluation results are depicted in Figure 2 and Table 1, which validate the performance of the OIS for spatially coherent spectral reconstruction without any electrical calibration. Figure 2(a) demonstrates that for ten randomly generated spectral amplitudes, the reconstruction results exhibit a good fit, with the output plane detectors’ intensities corresponding one-to-one with the spectral amplitudes and significantly exceeding the intensities of surrounding areas, indicating a high-quality energy distribution. Furthermore, as the size of the aperture increases, the reconstruction results show minimal variation, consistently achieving effective spectral reconstruction tasks. To further highlight the performance of the OIS, an input comprising solely of monochromatic light at 420 nm is considered, as shown in Figure 2(b). It is observed that the reconstruction results closely match the target intensity, with the relative intensities of other bands being significantly lower than that of the target. For wavelengths not seen during training, the output light intensity is mainly distributed in the detectors corresponding to the adjacent training wavelengths. For instance, for an input containing only the 405 wavelength band, the output light intensity is primarily distributed in the detectors corresponding to 400 nm and 410 nm. These results indicate that the OIS is capable of efficiently accomplishing the task of spectral reconstruction for spatially coherent inputs under discrete or continuous incident wavelengths.

**Table 1:** Spectral reconstruction performance comparisons between different neural network models. Spectral resolution, 10 nm. Evaluation metric, MSE (×10^−3^)/PCC (Pearson Correlation Coefficient).

Spectral reconstruction	Diffractive neural	Aperture size (μm^2^)			
Neural network model	Network size	160 × 160	320 × 320	400 × 400	800 × 800
Coherent reconstruction (optical)	800 × 800 × 2	6.96/0.9737	6.96/0.9737	6.97/0.9736	6.99/0.9735
Coherent reconstruction (optoelectronic)	800 × 800 × 2	0.26/0.9989	0.35/0.9984	0.35/0.9984	0.37/0.9983
Incoherent reconstruction (optical)	800 × 800 × 2	8.07/0.9705	8.64/0.9675	10.32/0.9597	18.46/0.9207
Incoherent reconstruction (optoelectronic)	800 × 800 × 2	0.50/0.9981	0.58/0.9978	0.75/0.9973	0.95/0.9969

### 3.2 Spatially incoherent optical reconstruction

**Figure 3:**
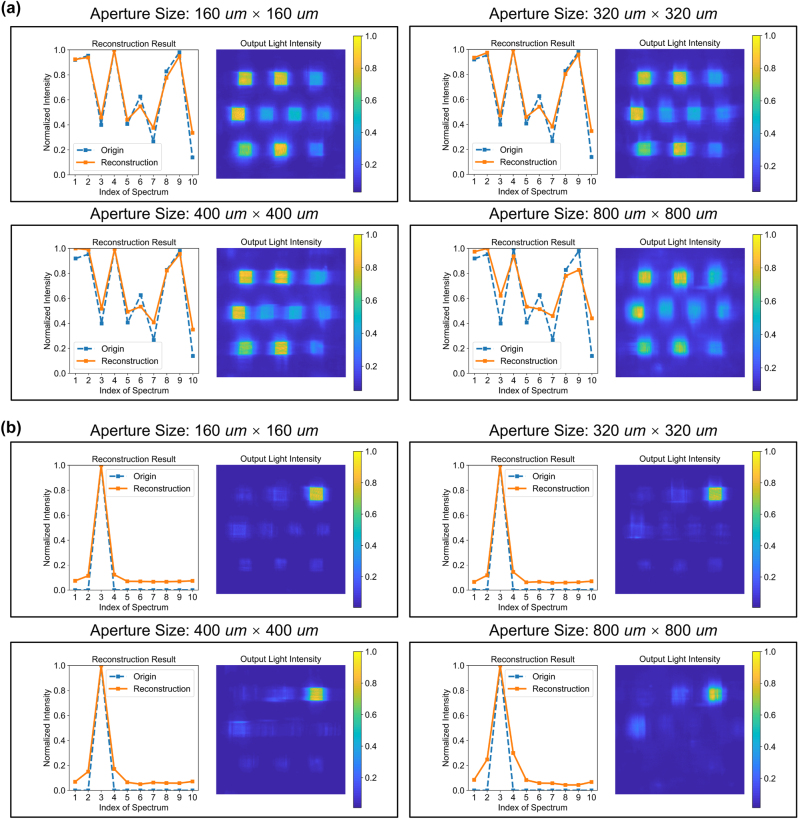
OIS for spatially incoherent spectral reconstruction without electric calibration. Spectral resolution, 10 nm. (a) Left: The spectral amplitude distribution of ten spectral bands randomly generated along with the results of spectral reconstruction. Right: The intensity distribution at the output plane. (b) Left: The spectral amplitude distribution of the input containing only the 420 nm wavelength band, along with the results of spectral reconstruction. Right: The intensity distribution at the output plane.

To elucidate the capability of the OIS in reconstructing spectra from spatially incoherent samples, we introduced random phases to the input plane to simulate the effect of spatial incoherence. The overall architecture remains unchanged, with the sole difference from the spatially coherent spectral reconstruction experiment being whether random phases are introduced to the input. The results, as shown in Figure 3(a) and Table 1, reveal that, in comparison to Figure 2(a), the reconstruction outcomes for the same spectral samples under incoherent conditions are inferior to those obtained under coherent conditions. Moreover, as the aperture size increases, the difficulty of reconstruction significantly escalates, leading to progressively worse outcomes. Similarly, as Figures 2(b) and 3(b) show, for inputs consisting of a single wavelength at 420 nm, the contrast of incoherent optical reconstruction results also falls short of coherent optical reconstruction outcomes. Nonetheless, overall, the OIS still demonstrates commendable capability in incoherent optical reconstruction, with the reconstruction results exhibiting a good correspondence with the inputs. This underscores the OIS’s generalized ability to handle both coherent and incoherent optical reconstruction challenges.

### 3.3 Spatially incoherent optical reconstruction with electric calibration

**Figure 4:**
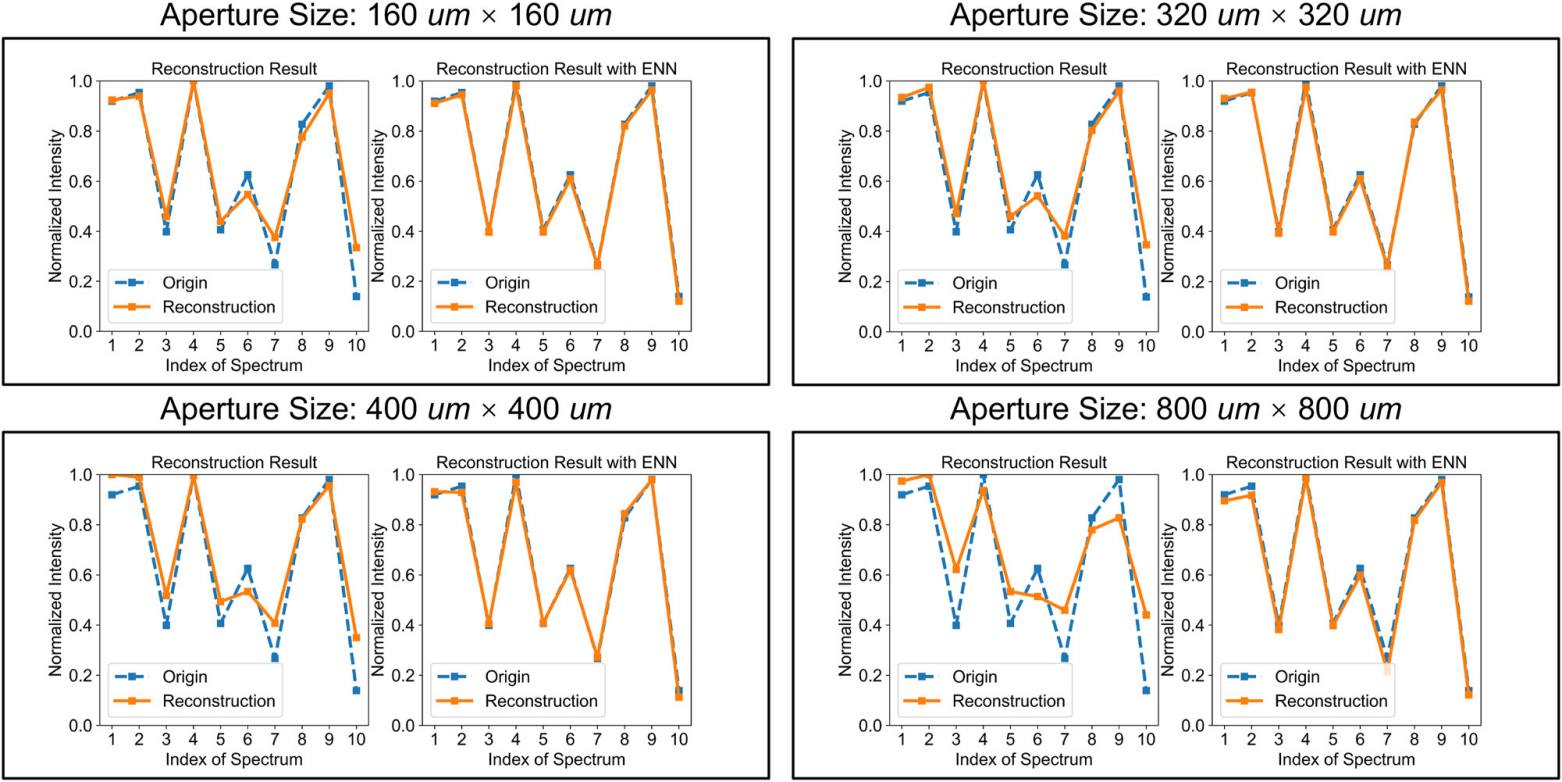
OIS for spatially incoherent spectral reconstruction with electric calibration. Spectral resolution, 10 nm. Left: The spectral amplitude distribution of ten spectral bands randomly generated along with the results of spectral reconstruction after the optical reconstruction module. Right: The spectral amplitude distribution of ten spectral bands randomly generated along with the results of spectral reconstruction after both the optical reconstruction module and the electrical correction module.

Utilizing the OIS, high-quality spectral reconstructions for spatially coherent inputs and slightly inferior reconstructions for spatially incoherent inputs can be achieved. To further enhance the OIS’s performance on spatially incoherent spectral reconstruction tasks, an optional electrical calibration module composed of electronic neural networks can be integrated following the optical reconstruction module for spectral calibration. Given the high quality of the initial reconstruction, a simple fully-connected neural network suffices for correction. The calibration results are shown in Figure 4 and Table 1. The left and right sides of Figure 4 display the reconstruction results before and after the electrical calibration module, revealing that post-electrical calibration, the reconstruction outcomes highly match the original inputs, with the quality even surpassing that of spatially coherent spectral reconstruction tasks without electric calibration. It is important to mention that electric calibration is also effective for coherent reconstruction, as shown in Table 1. These findings indicate that the electrical correction module can effectively complement the optical reconstruction module to achieve higher-quality reconstructions with minimal additional cost, thanks to the utilization of a shallow electronic neural network.

**Figure 5:**
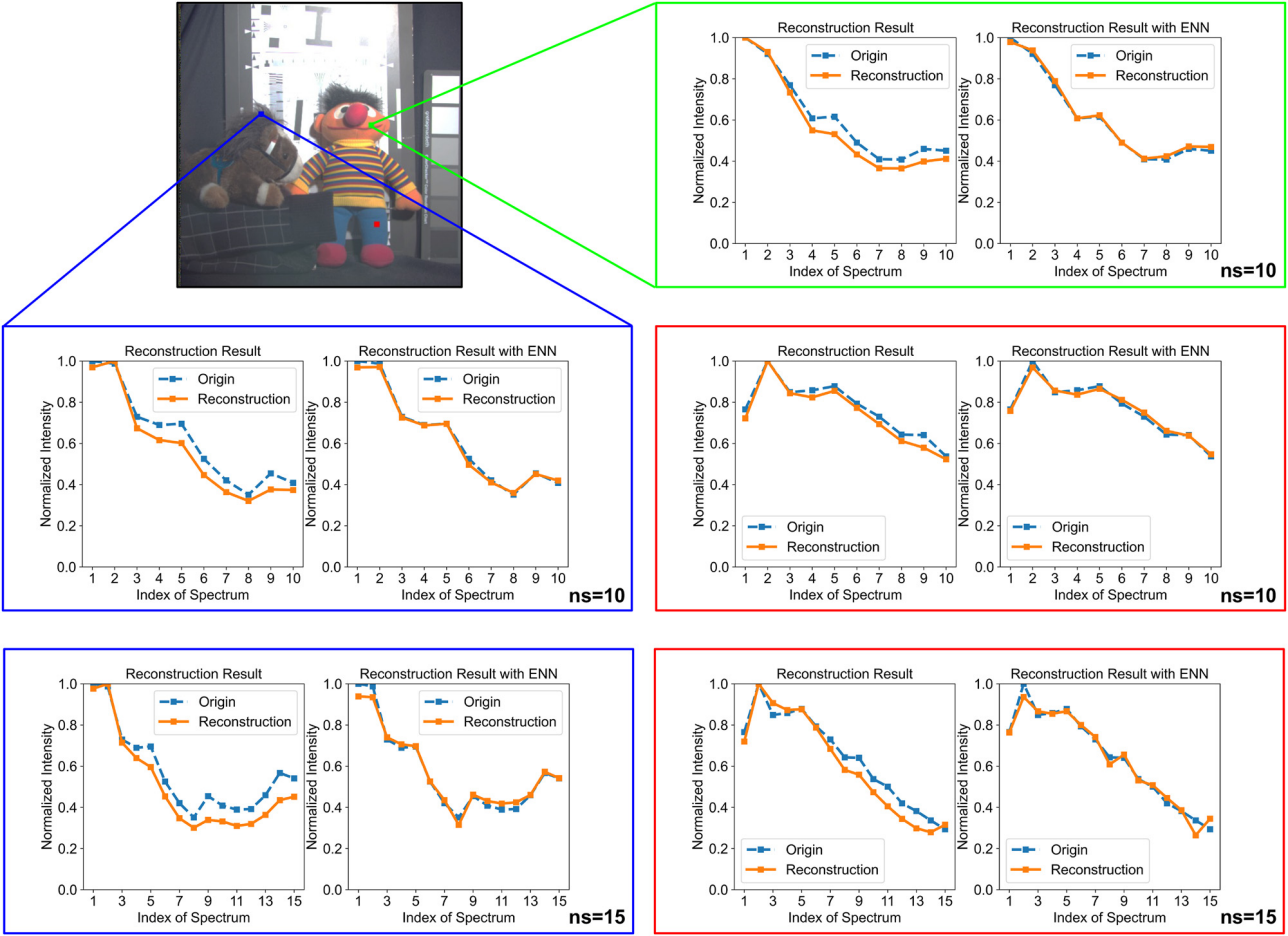
OIS for spatially incoherent spectral reconstruction with electric calibration in real-world database CAVE. Spectral resolution, 10 nm. ns, number of spectrum. On the “chart_and_stuffed_toy” sample, three pixels were randomly selected, showcasing the outcomes of optical reconstruction and electrical calibration.

To further validate the model’s generalization capabilities, experiments on spatially incoherent optical reconstruction with electrical calibration were conducted using the real-world dataset from the CAVE Multispectral Image Database [22]. This database contains a wide variety of real-world materials and objects, with a collection wavelength range of 400 nm–700 nm and a spectral resolution of 10 nm across 31 bands. For this work, a subset from 400 nm to 500 nm, covering 10 spectral bands, was extracted. Each image underwent testing by collecting data from three arbitrary pixels, employing a spatially incoherent OIS with an aperture size of 320 μm × 320 μm. As illustrated in Figure 5, the proposed model is capable of reconstructing the spectra for pixels, regardless of whether their spectral characteristics are similar or significantly different. The optical reconstruction outcomes are satisfactory and consistent with previous findings, and the electrical calibration module further enhances the quality of the reconstruction. These OIS architectures were trained on a custom dataset, and the model had not been exposed to the spectra of real-world objects before testing. Nonetheless, it still achieved commendable reconstruction results. This underscores the rationality of the proposed training methodology and demonstrates the robust generalization capability of the proposed architecture. Notably, the OIS is capable of handling a greater number of spectral bands. However, this capability requires more computational resources during training. Due to the current limitations in computational resources, we supplemented our experiments with 15-band cases, validated on the CAVE dataset. As shown in Table 2 and Figure 5, the 15-band cases successfully performed spectral reconstruction, although the reconstruction quality was somewhat lower compared to the 10-band experiments. This reduction in quality can be addressed by reducing the aperture size, increasing the number of layers, or the number of pixels per layer, which will be analyzed in the following discussion. Additionally, it is noteworthy that although the training time is considerable, once the OIS is fabricated, it can perform spectral reconstruction tasks at the speed of light.

**Table 2:** Performance comparison between 10-Band and 15-Band spectral reconstruction tasks. Spectral resolution, 10 nm. Aperture size, 320 μm × 320 μm. Evaluation metric, MSE (×10^−3^)/PCC.

Number of spectrum	Incoherent reconstruction (optical)	Incoherent reconstruction (optoelectronic)
10-Band	8.64/0.9675	0.58/0.9978
15-Band	16.72/0.9249	1.47/0.9945

## 4 Discussion

**Figure 6:**
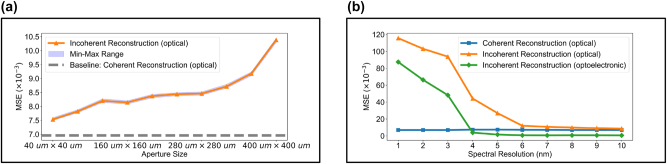
Impact of key parameters on OIS’s performance for spectral reconstruction. (a) Impact of aperture size for spatially incoherent spectral reconstruction without electronic calibration. (b) Impact of spectral resolution for different architectures.

For the task of reconstructing spatially incoherent spectra, we further analyze and examine the impact of the size of rectangular apertures. Without the addition of an electronic module, the results, as shown in Figure 6(a), indicate that the reconstruction error gradually increases with the size of the aperture. It is noteworthy that when the aperture size is getting small, the performance gradually approaches that of the spatially coherent spectral reconstruction using an aperture of 160 μm, 160 μm. We can hypothesize that a smaller aperture size, coupled with the reduced energy efficiency, leads to higher spatial coherence, approaching coherent light. In the intermediate region, the increase in error is relatively slow, making it an appropriate working area. For apertures of different sizes, we conducted ten tests using ten different random seeds. Overall, the minimal fluctuations in test results across different random seeds suggest that the design parameters for spatially incoherent spectra are well-chosen, with *N*_*tr*_ = 10 and *N*_*te*_ = 160 representing the number of repetitions for generating random phases during the training and testing processes, respectively. To minimize the leaked optical signals outside the diffractive layers, the OIS aperture limits signal leakage and the L-distribution loss enhances the signal-to-noise ratio. Other solutions include to use the physical sleeve encased with mirror to account for the leaked optical signals during the photonic computing.

To investigate the system’s scalability, we analyze the reconstruction errors across different spectral resolutions for a variety of architectures with the aperture size set to 320 μm × 320 μm, as illustrated in Figure 6(b). For architectures devoid of electronic correction, the spatially coherent optical reconstruction demonstrates low and consistent reconstruction errors throughout the 1–10 nm spectral resolution, facilitating high-quality spectral reconstruction even at the notably high spectral resolution of 1 nm. This efficiency markedly evidences the considerable superiority of this architecture in conducting precise spectral analyses. In spatially incoherent optical reconstructions without electronic correction, reconstruction errors are maintained at low levels in the spectral resolution range above 5 nm. However, with further increases in spectral resolution, reconstruction loss experiences a pronounced surge. The introduction of an electronic correction module at the backend significantly enhances reconstruction quality within the 4–10 nm range, at times even outperforming the spatially coherent reconstructions, albeit with comparatively elevated errors in resolutions below 4 nm, revealing the limitations of electronic correction capabilities. Overall, the designed architecture showcases high scalability, with coherent reconstruction without electronic correction achieving high-quality spectral reconstruction up to 1 nm, incoherent reconstruction without electronic correction achieving up to 6 nm, and incoherent reconstruction with electronic correction achieving up to 4 nm in high-quality spectral reconstruction.

**Figure 7:**
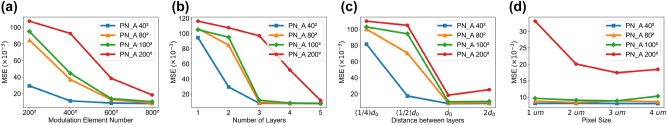
Impact of common parameters on OIS’s performance for spatially incoherent spectral reconstruction without electronic calibration. (a) Modulation element number. (b) Number of layers. (c) Distance between layers. (d) Pixel size. PN_A, the pixel number of aperture.

Finally, we investigated the impact of four common parameters in diffractive neural networks, with the results shown in Figure 7. Regarding the modulation element number, an increase in the modulation element number leads to a decrease in reconstruction loss. Concerning the number of layers, as the number of layers increases, the reconstruction loss decreases. It is noteworthy that for a 3-layer 200 × 200 OIS, the reconstruction loss is lower for the aperture’s pixel number of 40^2^, 80^2^, and 100^2^, but higher for the aperture’s pixel number of 200^2^. Meanwhile, a 5-layer 200 × 200 OIS maintains high reconstruction quality across different aperture’s pixel numbers, even for the aperture’s pixel number of 200^2^, which corresponds to scenarios without a rectangular aperture. In terms of the distance between layers, the least reconstruction loss corresponds to distances calculated based on the maximum half-cone diffraction angle theory, thereby affirming the rationale behind the experimental distance selection. As for pixel size, while the aperture’s pixel number of 40^2^, 80^2^, and 100^2^ show minor loss variations across different pixel sizes, the aperture’s pixel number of 200^2^ presents relatively greater loss with smaller pixel sizes.

## 5 Conclusions

In this study, we have demonstrated the capability of OIS in performing spectral reconstructions with remarkable accuracy for both spatially coherent and incoherent light sources. This achievement validates the potency of the optical intelligence architecture, with the assistance of an auxiliary optional electrical module, marking a paradigm shift towards high-fidelity spectral reconstructions. The careful consideration of spatial and partial temporal coherence in our proposed framework solidifies its status as a pivotal reference for deploying photonics neural networks in the spectral analysis of real-world objects. The all-optical diffractive neural network, complemented by the simplicity of the electrical module, makes our architecture exceptionally efficient, facilitating rapid inference and reducing system complexity. Looking forward, building on advancements in spectral imaging [23], [24], we aim to expand the application of OIS by transitioning from single-point spectral reconstruction to high-speed, high-quality spectral imaging. Our endeavors will pivot towards broadening the spectral bandwidth and refining spectral resolution, aiming to achieve a more nuanced spectral understanding in various applications.
